# Reactive oxygen species drive wound epithelium and blastema formation in regenerating tail of *Scincella tsinlingensis*

**DOI:** 10.1186/s12983-026-00617-2

**Published:** 2026-06-02

**Authors:** Yiwen Zhang, Jinyu Sun, Xinyue Wang, Yuwei Gao, Qing Li, Chun Yang

**Affiliations:** School of Life Sciences, Shanxi Normal University, Taiyuan, Shanxi 030031 China

**Keywords:** *Scincella tsinlingensis*, Tail regeneration, Reactive oxygen species, Hydrogen peroxide

## Abstract

Hydrogen peroxide (H_2_O_2_) is a crucial signaling molecule in vertebrate regeneration, yet its functional role across diverse lizard species remains incompletely defined. This study elucidates the master regulatory function of H_2_O_2_ during tail regeneration in the lizard *Scincella tsinlingensis* using integrated pharmacological, morphological, histological, and transcriptomic approaches. Pharmacological modulation revealed that H_2_O_2_ markedly enhances regeneration, achieving 5.89 ± 0.69 mm tail length at 21 days post-amputation (dpa), whereas ROS inhibition severely impairs regenerative outgrowth (0.88 ± 0.07 mm). Transcriptomic analysis at 7 dpa demonstrated that H_2_O_2_ establishes fundamentally distinct molecular trajectories, with only 5 common differentially expressed genes (DEGs) shared between the DMSO-vs-APO (713 DEGs) and DMSO-vs-H_2_O_2_ (593 DEGs) comparisons. Functionally, H_2_O_2_ synchronizes three core pathways—phagocytosis, immune regulation, and cellular motility—into an integrated repair program while temporally coordinating MAPK, mTOR, Wnt, and Hippo-YAP signaling cascades. This establishes a sequential regulatory framework where the immediate post-amputation period constitutes an H_2_O_2_-dependent critical window with limited plasticity, explaining the only partial rescue achieved with delayed intervention. Transcriptomic data further suggest inferred associations of NADPH oxidase 2/DUOXA2-derived H_2_O_2_ with cellular proliferation, YAP1 expression, potential EMT (via HIF-1α), as well as G2/M arrest and chromatin remodeling. These findings support the paradigm that ROS act as evolutionarily conserved, spatiotemporal integrators of tissue repair, coordinating injury response, immune modulation, metabolic reprogramming, and developmental pathway activation. This work provides a mechanistic foundation for developing redox-based therapies aimed at enhancing regeneration in clinical contexts of impaired healing.

**Supplementary Information:**

The online version contains supplementary material available at 10.1186/s12983-026-00617-2.

## Background

Reactive oxygen species (ROS), including non-radical components such as hydrogen peroxide (H_2_O_2_), are primarily generated through NADPH oxidases (Noxes) and the mitochondrial electron transport chain, where superoxide anions are converted to H_2_O_2_ via dismutation or catalysis by superoxide dismutase [1–3]. Cells maintain ROS homeostasis through antioxidant systems such as superoxide dismutase (SOD) and peroxiredoxins (Prx), while appropriate concentrations of ROS create an “oxidative stress” state that plays a key role in cellular signaling [4–6]. As a diffusible signaling molecule, H_2_O_2_ can activate pathways such as MAPK and PI3K/Akt, as well as transcription factors including HIF-1α and NF-κB [6, 7]. Recent studies in mammalian systems have further defined the therapeutic potential of modulating ROS, such as through biomaterials that target ROS to activate the PI3K/Akt pathway for bone repair [8] or strategies that fine-tune ROS to promote stem cell function and angiogenesis in diabetic complications [9, 10].

It has been shown to promote appendage or tail regeneration in various animal models, such as zebrafish [11, 12], Xenopus [13], and axolotl [14], by activating NADPH oxidases or functioning as a redox signal [15–18]. During regeneration, ROS regulate key cellular events through multiple signaling pathways. In the Hippo-YAP pathway, H_2_O_2_ upregulates NEDD4 to promote LATS1/2 degradation [19, 20], or via the PP2A regulatory subunit STRN3 [21], inhibits LATS1/2, thereby activating YAP/TAZ and promoting processes such as epithelial-mesenchymal transition (EMT) [22, 23]. In the PI3K/AKT pathway, ROS enhance receptor tyrosine kinase (RTK) signaling by oxidatively inhibiting phosphatases such as PTP1B, or by inactivating PTEN, thereby activating the PI3K-Akt axis to promote cell growth and proliferation [24]. This is exemplified in regenerative contexts such as photobiomodulation, where ROS-induced PI3K/Akt activation drives stem cell differentiation [22, 25]. Furthermore, under hypoxic conditions, ROS stabilize HIF-1α, which upregulates Twist1 to induce EMT. The Twist1 target BMI-1 [26, 27], a key component of the chromatin remodeling complex PRC1, is essential for maintaining stem cell properties and blastema formation [28–30]. Akin to this, pathological oxidative stress in conditions like osteoporosis can be countered by activating antioxidant pathways such as Nrf2/HO-1/GPX4 to inhibit ferroptosis and promote bone healing [31, 32], highlighting the conserved yet context-dependent nature of redox regulation.

Lizards are well-established amniote models for studying tissue regeneration [33], with extensive research documenting their regenerative capabilities [34–36]. Among them, the Chinese endemic ovoviviparous lizard, *Scincella tsinlingensis*, has been shown to possess notable neurobiological features, including defined telencephalon cytoarchitecture [37] and seasonally regulated cell proliferation [38], as well as remarkable regenerative traits such as scar-free cutaneous wound healing [39], tail regeneration with distinct morphological restructuring [40], and active neurogenesis within the regenerating tissue [41]. Given the limited regenerative capacity of mammals, deciphering the molecular mechanisms underlying lizard tail regeneration holds significant translational potential. In this context, reactive oxygen species (ROS) have emerged as key spatiotemporal regulators that integrate multiple signaling pathways—including MAPK, mTOR, Wnt, and Hippo-YAP—in a manner analogous to their coordinating roles in mammalian tissue repair processes such as cardiac [42], neural [43], and lung regeneration [44]. This study therefore focuses on the central role of ROS during blastema formation in *S. tsinlingensis*, employing transcriptomic approaches to identify critical regulatory targets. Our aim is to elucidate the regulatory network governing tail regeneration and to provide a theoretical foundation for regenerative medicine that bridges insights from reptilian models to therapeutic strategies in mammals.

## Methods

### Lizards

Adult *S. tsinlingensis* (snout-vent length 4.8–5.7 cm) were collected from the Qiliyu forest area in the Taiyue Mountains, Shanxi, China (36°21′–36°45′N, 110°40′–112°21′E). All procedures were conducted in accordance with the National Institutes of Health Guide for the Care and Use of Laboratory Animals (8th edition, 2011) and approved by the Animal Ethics Committee of Shanxi Normal University (Approval No. SXNU-STEC-2024-0316). Lizards were housed in soil-lined terraria under controlled conditions: 40%–60% humidity, with a 10-h light/24°C day and 14-h dark/19°C night cycle, and provided with *Tenebrio molitor* larvae and water ad libitum, following established husbandry protocols [40, 41].

### Drug treatments

Tail autotomy was induced at 2 cm posterior to the cloaca. After autotomy, lizards were individually housed and monitored. Drug treatments were adapted from Zhang et al. (2016) [46]. The tail stump was immersed in an Eppendorf tube containing 50 µM H_2_O_2_ (Xiongda Chemical Co., Ltd., Maoming City, China) or 600 µM apocynin (APO, Santa Cruz, SC203321; a NADPH oxidase inhibitor, Sigma-Aldrich) dissolved in 0.1% dimethyl sulfoxide (DMSO, Bio Basic Inc.) saline solution for 6 h. Subsequently, 5 µl of 50 µM H_2_O_2_ or 600 µM APO were administered via intraperitoneal injection every two days. Control experiments were performed following the same protocol, with an equivalent amount of vehicle.

**Fig. 1:**
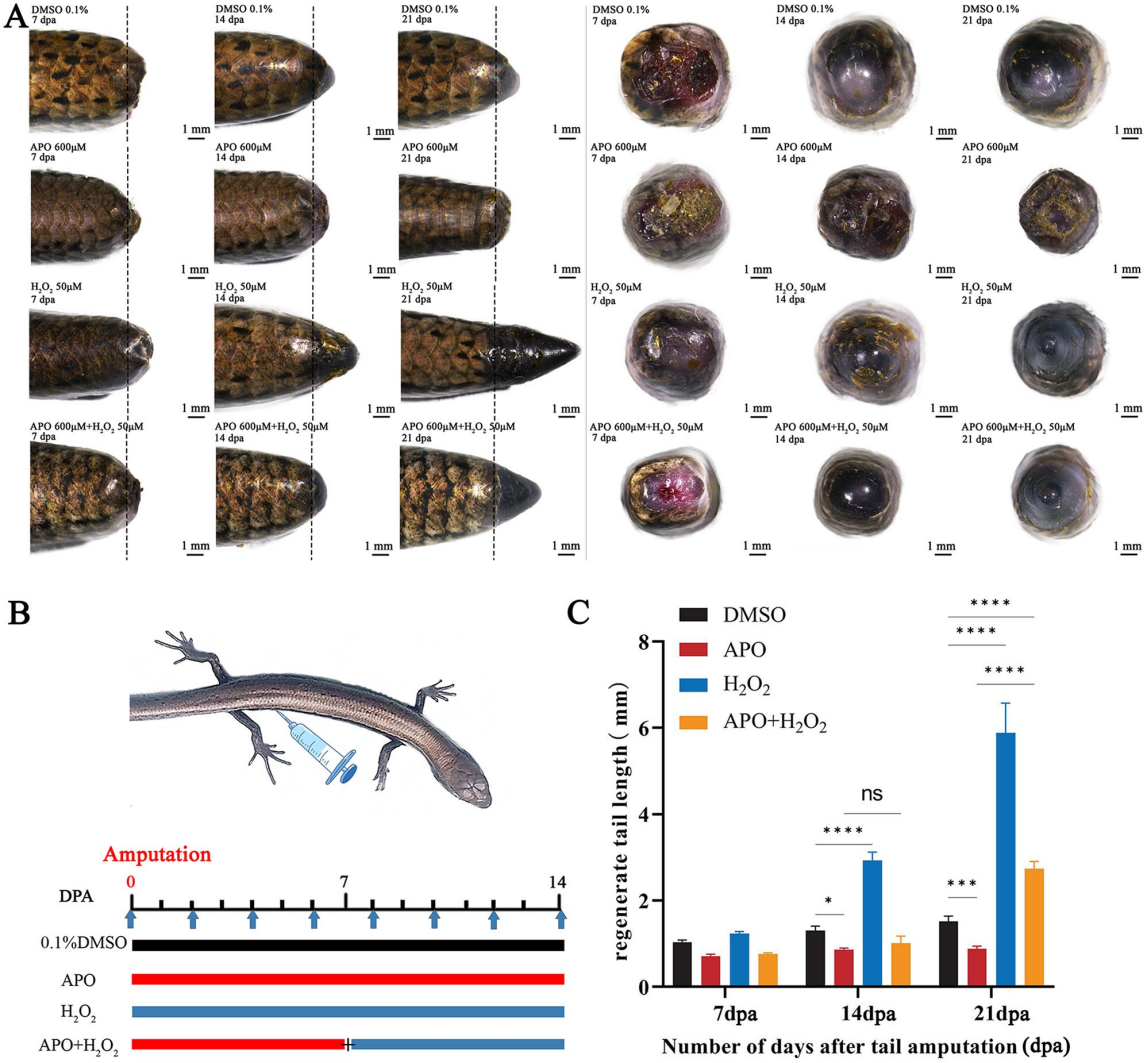
Pharmacological modulation of ROS levels affects tail regeneration in *S. tsinlingensis.* (**A**) Morphological observation of regenerated tails following DMSO, Apocynin (APO) or H_2_O_2_ administration. (**B**) Schematic of intraperitoneal injection protocol post-autotomy. Quantification of regenerated tail length across experimental groups (*n* = 6 for each group). Following tail amputation, DMSO, APO, or H_2_O_2_ were administered every other day for 14 dpa. In the rescue group, APO was injected during 0–7 dpa followed by H_2_O_2_ during 7–14 dpa. (**C**) H_2_O_2_ rescues and enhances tail regeneration length over time. H_2_O_2_ significantly increased regeneration length by 14 and 21 dpa. The rescue group showed intermediate regeneration, significantly surpassing the inhibited APO group by 21 dpa, indicating that H_2_O_2_ effectively mitigates ROS-inhibition effects

Four experimental groups received intraperitoneal injections as follows: a DMSO vehicle control group; an APO-treated group (Apocynin); an H_2_O_2_-treated group to enhance ROS; and a rescue group, which was sequentially treated with APO during 0–7 days post-amputation (dpa) and with H_2_O_2_ during 7–14 dpa. Regenerated tail tissues were collected at designated time points via secondary amputation (Fig. 1B) and processed for subsequent histological, immunohistochemical, and molecular analyses.

### Histology and immunohistochemistry

The collected samples (*n* = 3 for each group) were fixed in 4% paraformaldehyde at 4 °C for 3 days and subsequently decalcified in Leica Surgipath Decalcifier I with constant shaking for an additional 3 days. A portion of these tail samples was then dehydrated through a graded ethanol series, cleared in xylene (three changes, 1 h each), and embedded in paraffin wax. Serial 10 μm-thick sections were prepared and mounted on polylysine-coated glass slides for hematoxylin-eosin (H.E.) staining. The remaining samples were post-fixed in the same fixative overnight at 4 °C, cryoprotected by immersion in sequentially 15% and 30% sucrose solutions, and finally embedded in OCT compound. Longitudinal sections of 25 μm thickness were obtained using a freezing microtome (CM-1850, Leica, Germany) and stored at -20 °C for subsequent ROS detection.

Paraffin sections were dewaxed in xylene and rehydrated through a graded ethanol series, followed by endogenous peroxidase blockade with 3% hydrogen peroxide at 25 °C for 25 min in the dark and three 5-min PBS washes. After antigen retrieval for 30 min and subsequent washing, the sections were permeabilized with 0.2% Triton X-100 for 10 min at room temperature, drained, and blocked with 5% BSA for 30 min. The blocking solution was then removed, and sections were incubated overnight at 4 °C with the following primary antibodies: Twist1 (Beyotime, AF8274, 1:100), Slug (Beyotime, AF7998, 1:100), BMI-1 (Beyotime, AF0072, 1:100), EZH2 (Beyotime, AF6828, 1:100), ROR2 (Beyotime, AF7905, 1:100), WNT5A (Beyotime, AF8355, 1:100), HIF-1α (Beyotime, AG2135, 1:100), PHH3 (Ser10) (Beyotime, AF1180, 1:200), Caspase 3(p12) (Boster, BM4620, 1:100), YAP1 (Boster, PB1021, 1:400). The next day, sections were warmed to room temperature, washed three times, and incubated in the dark at 37 °C for 1 h with a fluorescently conjugated secondary antibody (Boster, SABC-DyLight Cy3, SA1074/SA1072). After five additional washes in the dark, nuclei were counterstained with DAPI for 20 min, followed by a final wash and mounting with an anti-fade medium.

### ROS assay

To evaluate the role of ROS during tail regeneration in *S. tsinlingensis*, endogenous ROS levels were modulated and assessed at 7, 14, and 21 dpa, followed by morphological and histological examination. Intracellular ROS was detected using the fluorescent probe DCFH-DA (Sigma, D6883), which is hydrolyzed to DCFH and then oxidized by ROS into the fluorescent product DCF; the resulting green fluorescence intensity is proportional to ROS levels [45]. Regenerating tail tissues collected at each time point were incubated in the dark with 50 µM DCFH-DA for 30 min. After staining, samples were protected from light throughout paraffin embedding. Sections were prepared, dried, deparaffinized, and rehydrated, then mounted with an anti-fade medium for fluorescence imaging.

### Transcriptome sequencing analysis

#### Tissue sample collection

Forty-eight healthy adult *S. tsinlingensis* of comparable size were randomly assigned to four groups (*n* = 12 for each group): three groups underwent 7 dpa drug treatments, while one served as the 0 dpa control. After anesthesia by refrigeration at 4 °C, initial tail autotomy was performed 2 cm posterior to the cloaca. Following the 7 dpa treatment regimen, a secondary autotomy was conducted to harvest 7 dpa regenerated tissues. These samples, together with 0 dpa specimens from the initial amputation, were snap-frozen in liquid nitrogen and maintained at -80 °C for subsequent analysis.

### RNA extraction and quality control

RNA quality was assessed using a NanoDrop 2000 spectrophotometer for purity/concentration and an Agilent 2100 Bioanalyzer for integrity. Three qualified RNA samples per group were subsequently sent to Biomarker Technologies Corporation (Beijing, China) for cDNA library construction and sequencing.

### cDNA library construction and Illumina sequencing assembly

Following mRNA purification and fragmentation, double-stranded cDNA was synthesized, end-repaired, adenylated, and ligated with NEBNext adapters. Library fragments were purified using the AMPure XP system (Beckman Coulter, Beverly, USA), PCR-amplified, and repurified with the same system. Library quality was verified on an Agilent Bioanalyzer 2100, followed by sequencing on the Illumina HiSeq 2000 platform. After quality control, clean data were subjected to de novo assembly via Trinity software to construct a Unigene library.

### Gene annotation and differential expression gene analysis

Unigenes were annotated against multiple databases using DIAMOND software, including NR, KOG/COG/eggnog, Swiss-Prot, and KEGG. KEGG and GO Orthology analyses were performed with KOBAS and InterProScan, respectively, while HMMER was employed for Pfam alignment. Differential expression was identified using thresholds of |Fold Change| ≥ 1.5 and *p*-value < 0.01.

### Enrichment analysis of differential expressed genes (DEGs)

Gene Ontology (GO) enrichment analysis of DEGs was performed using topGO R software package, employing the Kolmogorov-Smirnov test. Statistical enrichment of DEGs in KEGG pathways was analyzed using KOBAS software.

### Quantitative real-time PCR (qRT-PCR)

RNA quality was assessed by A260/A280 ratio using a Scandrop 100 analyzer. cDNA was synthesized with the TUREscript 1st Stand cDNA SYNTHESIS Kit (Aidlab) in 20 µl reactions and stored at -80 °C. Using primers listed in Table 1, qPCR reactions were centrifuged at 6000 rpm for 30s at 4 °C, then amplified on an Analytik Jena-Easycycler with: 95 °C for 3 min; 39 cycles of 95 °C/10s and 60 °C/30s.

**Table 1:** The sequence of primers

Gene	Forward (5’-3’)	Reverse (5’-3’)
*akt1*	F: ACATTAAGCCGTCCCACT	R: CACCGCACAGATGATTACAAT
*mtor*	F: AGAGGACTTGCTGCTGTA	R: GTCAGTATGCGTGCCTATT
*hif1a*	F: CGAGAAGAAGCGGATTAGT	R: CGTAGAACACCTCAGACTC
*twist1*	F: AGACTCGGCACTTGGATT	R: GTCTCTCGCCTGAAATGC
*wnt5a*	F: TTGTTGTGGAGGTTCATCAG	R: GACTATGGCTACCGCTTTG
*nadph oxidase 2*	F: GTGGTGAAGGCTACATAGA	R: AGAGTGGTTGGTGAATGAA
*strn3*	F: GCCCTAATGTGGACCCTTA	R: CTGAGCAAGATAGCAAGTGATT
*tead1*	F: AAAGCCAGCCAAATCTCA	R: TCGCCAAGAAGGAATAAGTT
*gapdh*	F: TGCACCACCAACTGCTTAGC	R: GGCATGGACTGTGGTCATGA

### Cellular observation and documentation

Gross anatomy was documented with a stereomicroscope. Histological sections (H&E) were imaged using a BX-40 microscope, while fluorescence sections (ROS and immunofluorescence) were visualized with a BX-53 microscope. Image processing and figure assembly were performed in Adobe Photoshop CC.

### Statistical analysis

Three randomly selected fluorescence sections  (200× magnification) per group were analyzed. After uniform brightness/contrast adjustment in Photoshop, the average fluorescence intensity in wound and dermal regions was quantified with Image J. Data are expressed as mean ± SD. Statistical comparisons for fluorescence intensity and tail length used two-way ANOVA with multiple comparisons; qRT-PCR data were analyzed by one-way ANOVA (GraphPad Prism 9). Significance levels: *p* < 0.05, *p* < 0.01, *p* < 0.001, *p* < 0.0001; ns, not significant.

## Results

### H_**2**_O_**2**_-mediated ROS signaling promotes blastema formation and tail regeneration in *S. tsinlingensis*

To elucidate the functional role of ROS in tail regeneration of *S. tsinlingensis*, regeneration phenotypes were examined under modulated ROS conditions at 7, 14, and 21 days post-amputation (dpa) (Fig. 1A-C). Experimental groups included control, APO-treated (ROS inhibition), H_2_O_2_-treated (ROS enhancement), and a rescue group (APO during 0–7 dpa followed by H_2_O_2_ during 7–14 dpa) (Fig. 1B). Morphological analysis revealed that controls progressed through normal regeneration stages - scab formation and wound epithelium at 7 dpa, apical ectodermal cap and early blastema by 14 dpa, and late blastema at 21 dpa - ROS inhibition via APO resulted in delayed wound epithelialization at 7 dpa, accompanied by persistent accumulation of cells near the wound edge that did not resolve by 21 dpa, at which time control and rescue groups exhibited an elongated blastema, as well as delayed tissue remodeling by 14 dpa, and complete wound epithelialization only by 21 dpa. Conversely, H_2_O_2_ treatment accelerated regeneration, achieving wound closure by 7 dpa, advanced blastema by 14 dpa, and scale regeneration by 21 dpa. The rescue regimen showed intermediate progression with persistent wound clots at 7 dpa but achieved AEC formation by 14 dpa and early blastema by 21 dpa (Fig. 1A). Regeneration length quantification corroborated these findings: at 7 dpa, the H_2_O_2_ group showed the longest regeneration (1.23 ± 0.05 mm) though without significant differences among groups; by 14 dpa, the H_2_O_2_ group (2.93 ± 0.20 mm) significantly exceeded others, with the rescue group (1.02 ± 0.16 mm) intermediate between control (1.31 ± 0.10 mm) and APO groups (0.86 ± 0.04 mm); at 21 dpa, the H_2_O_2_ group achieved maximum length (5.89 ± 0.69 mm) while the rescue group (2.74 ± 0.17 mm) significantly surpassed both control (1.51 ± 0.13 mm) and APO groups (0.88 ± 0.07 mm) (Fig. 1C). Collectively, these results demonstrate that H_2_O_2_ enhances tail regeneration and partially rescues APO-induced regeneration impairment, while ROS suppression persistently inhibits regenerative growth.

### H_2_O_2_ restores blastema formation and epithelial morphogenesis in ROS-deficient tail regeneration

**Fig. 2:**
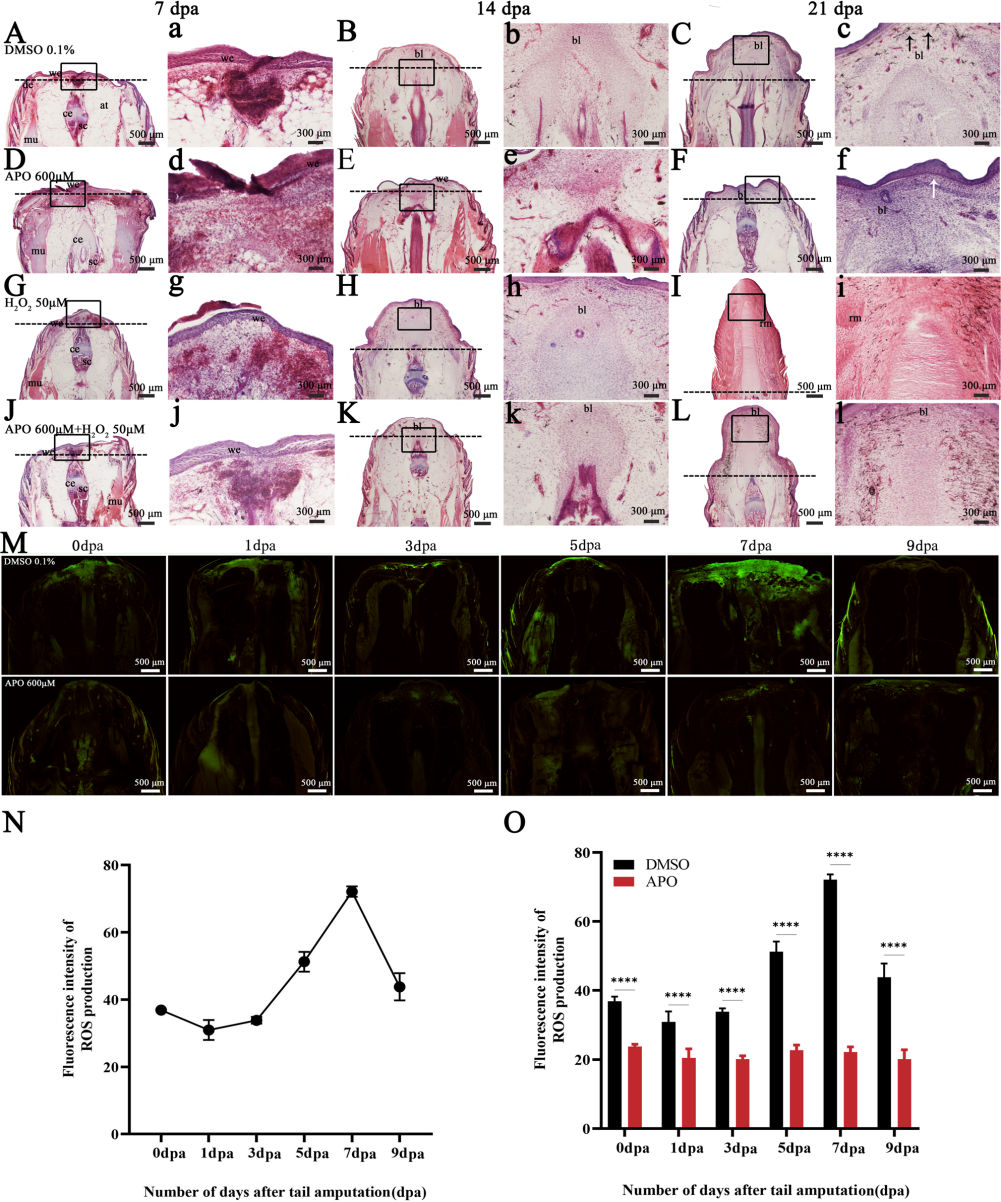
Histological and redox dynamics during tail regeneration under redox perturbation. (**A**–**L**) H&E-stained tail sections from different treatment groups; corresponding higher-magnification views (a–l) show boxed regions. (**M**) H_2_DCFDA-based quantification of ROS levels before and after APO treatment reveals amputation-induced redox changes. (**N**) Temporal dynamics of ROS production during regeneration. (**O**) Statistical analysis of ROS fluorescence intensity (*n* = 3 for each group; *p* < 0.0001 between DMSO and ROS inhibitor groups). Dashed line indicates the amputation plane. Arrows indicate undifferentiated cell clusters in loose matrix. Abbreviations: at, adipose tissue; bl, blastema; ce, central vertebra; de, dermis; mu, muscle tissue; rm, regenerated muscle; sc, spinal cord; we, wound epithelium. Data are mean ± SD

Histology of tail regeneration in *S. tsinlingensis* demonstrated distinct regenerative responses under modulated ROS conditions (Fig. 2A-L). Control specimens exhibited complete wound closure by epithelial migration at 7 dpa (Fig. 2A), epithelial thickening with early blastema formation at 14 dpa (Fig. 2B), and undifferentiated cell clusters in loose matrix at 21 dpa (Fig. 2C). ROS inhibition via APO resulted in impaired epithelialization at 7 dpa (Fig. 2D), absence of a blastema despite complete re-epithelialization at 14 dpa (Fig. 2E), and by 21 dpa, blastema cells had accumulated and formed a discrete boundary with the underlying muscle tissue (Fig. 2F). Conversely, H_2_O_2_ treatment accelerated regeneration, achieving complete wound closure by 7 dpa (Fig. 2G), robust blastema formation by 14 dpa (Fig. 2H), and advanced structures including regenerating cartilage surrounded by new muscle at 21 dpa (Fig. 2I). The rescue regimen showed partial recovery with early blastema accumulation at 14 dpa (Fig. 2K) and thickened epithelium with defined blastemal boundaries at 21 dpa (Fig. 2L), indicating that H_2_O_2_ can partially overcome ROS suppression-mediated impairment of blastema formation.

### ROS production is dynamically regulated during tail regeneration

ROS production was dynamically monitored following tail amputation (Fig. 2M-O). At 0 dpa, high ROS levels were detected at the wound site (Fig. 2M). ROS signals increased by 3 and 5 dpa, fluorescence accumulation was observed not only in the wound area but also in muscle tissues. ROS expression peaked in the blastema region at 7 dpa, coinciding with the active phase of cell proliferation, and declined by 9 dpa (Fig. 2N). In APO-treated groups, quantitative analysis confirmed significant reduction of ROS levels compared to controls (Fig. 2O), demonstrating effective ROS suppression and suggesting potential disruption of redox homeostasis during regeneration.

### De novo transcriptome assembly and functional annotation of regenerating tail tissue

RNA samples were collected from regenerating tail tissues of *S. tsinlingensis* at 7 days post-amputation, including control (DMSO), APO-treated, and H_2_O_2_-treated groups, along with 0-day control tissues. All twelve samples met the requirements for subsequent transcriptome sequencing. A total of 80.53 Gb of clean data was obtained, with each sample yielding ≥ 6.20 Gb. All samples exhibited Q30 base percentages ≥ 94.20% and GC contents ranging from 47.77% to 48.82% (Table S1). De novo assembly generated 183,658 transcripts and 99,121 unigenes, with mean lengths of 1,513.42 bp and 1,026.6 bp, respectively. Among these, 25,133 unigenes exceeded 1 kb in length, and the unigene N50 was 2,253 bp (Table S2).

**Fig. 3:**
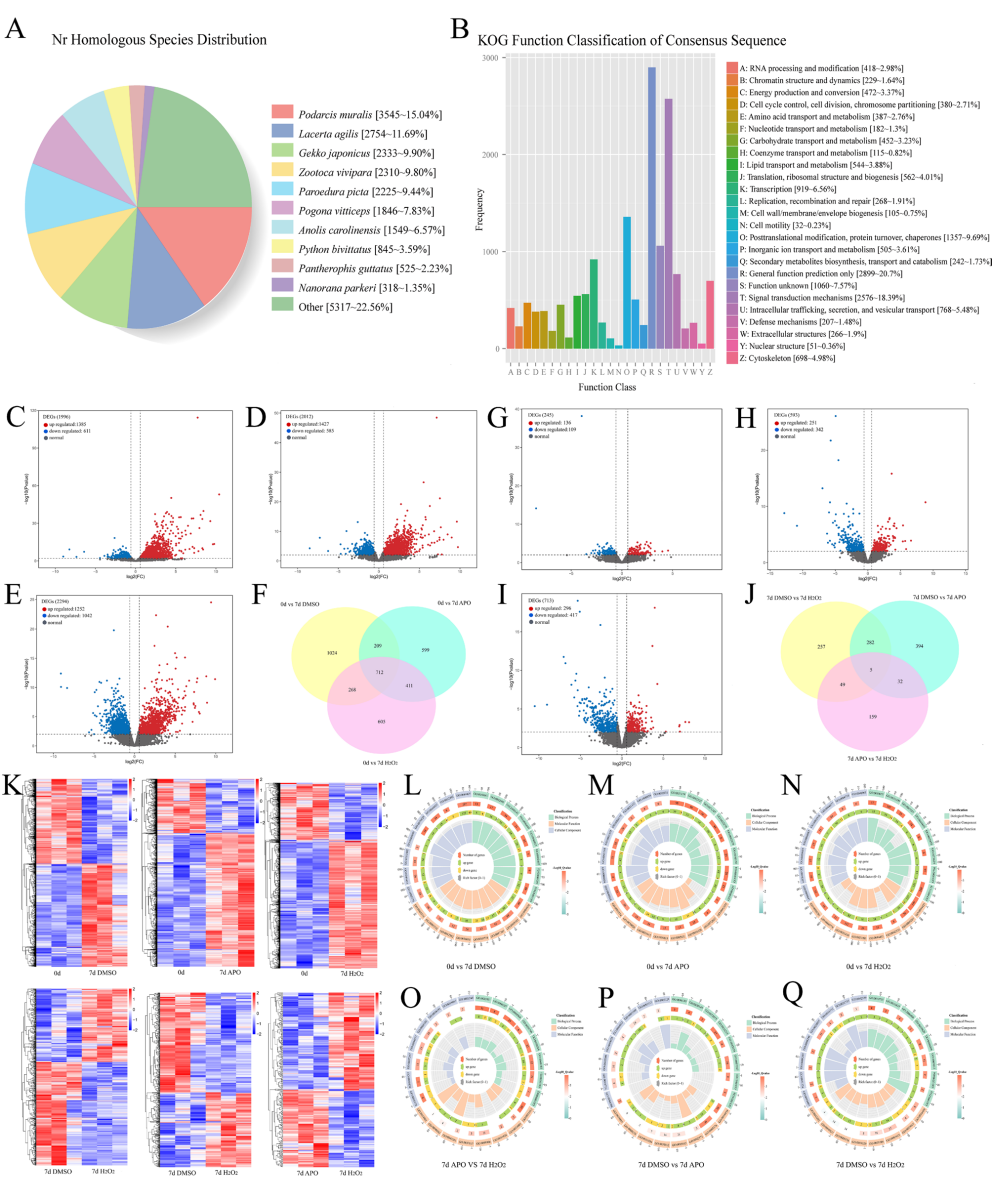
Transcriptome dynamics during regeneration under redox modulation. (**A**) Taxonomic distribution of annotated unigenes. (**B**) Functional classification of unigenes by KOG categories. (**C**–**E**) Volcano plots of DEGs between 0 dpa and each 7 dpa treatment group (DMSO, APO, H_2_O_2_). (**F**) Venn diagram showing high APO–H_2_O_2_ overlap among temporal DEGs (712 shared genes). (**G**–**I**) Volcano plots of DEGs from pairwise comparisons of 7 dpa treatments. (**J**) Venn diagram of treatment-specific DEGs (5 common genes). (**K**) Heatmap of DEGs across all groups. (**L**–**Q**) Enriched biological processes: H_2_O_2_ upregulates immune-metabolic pathways; APO suppresses regenerative programs

Following DIAMOND alignment, 26,749 unigenes (26.99% of total) were successfully annotated (Table S3). The highest annotation rate was achieved in the NR database (23.78%), followed by eggNOG (21.54%) and GO (20.12%). KEGG and Pfam annotations covered 19.60% and 17.93% of unigenes, respectively, while KOG, Swiss-Prot, and COG accounted for 14.13%, 11.29%, and 4.56%, respectively. Nr database homologous sequence results showed that the highest number of matches was for *Podarcis muralis* (3545 unigenes), followed by *Lacerta agilis* (2754), *Gekko japonicus* (2333), *Zootoca vivipara* (2310), and *Paroedura picta* (2225) (Fig. 3A). In total, 14,006 unigenes were added to the KOG database and categorized into 25 different KOG categories (Fig. 3B). The largest group of genes was categorized as being involved in general function (2899 unigenes (20.7%)), followed by signal transduction pathways (4169 unigenes (14.28%)) and posttranslational modification, protein turnover, and chaperones (2576 unigenes (18.39%)). Cell motility was the least represented group (32 unigenes (0.23%)).

### Distinct transcriptional trajectories are dictated by ROS modulation

To identify genes involved in ROS signaling and blastema development, we conducted transcriptome sequencing of tail tissues at 0 dpa and regenerating tails at 7 dpa under pharmacological treatments. Volcano plots visually represent the expression divergence between the 0 d and each 7 d treatment group (DMSO, APO, H_2_O_2_) (Fig. 3C-E). Subsequent analysis identified a total of 3,909 DEGs after removing duplicates. The comparison of 0 d vs. 7 d DMSO yielded the highest number of DEGs (2,294), followed by 0 d vs. 7 d APO (2,012) and 0 d vs. 7 d H_2_O_2_ (1,996). Pairwise overlaps revealed that the APO and H_2_O_2_ comparison shared the most DEGs (1,123), while 712 genes were common to all three comparisons, as detailed in the Venn diagram (Fig. 3F).

As visualized in the volcano plots (Fig. 3G-I), transcriptomic analysis revealed distinct expression patterns across the 7 d treatment groups, leading to the identification of 1,178 non-redundant DEGs. The number of DEGs was highest for the 7 d DMSO vs. 7 d APO comparison (713), compared to 593 for 7 d DMSO vs. 7 d H_2_O_2_ and 245 for 7 d APO vs. 7 d H_2_O_2_. Strikingly, the overlap between these comparisons was limited, with only 5 genes common to all three (Fig. 3J). Specific pairwise overlaps included 287 DEGs shared between DMSO vs. APO and DMSO vs. H_2_O_2_, 37 between DMSO vs. APO and APO vs. H_2_O_2_, and 54 between DMSO vs. H_2_O_2_ and APO vs. H_2_O_2_. This pattern suggests that DMSO and H_2_O_2_ treatments maintain expression of regeneration-related genes, whereas APO substantially disrupts the regenerative program. Differentially expressed genes (DEGs) in each comparison group were screened, and hierarchical clustering analysis was performed on the screened DEGs. Genes with identical or similar expression patterns were clustered, and the results are shown in Fig. 3K.

### Cluster analysis defines redox intervention-specific transcriptional responses

Clustering of DEGs delineated distinct expression patterns across experimental groups (Fig. 3L-Q). Compared with 0 dpa controls, all groups at 7 dpa exhibited predominant upregulation: DMSO (1,252 up/1,042 down), APO (1,427 up/585 down), and H_2_O_2_ (1,385 up/611 down). In contrast, pairwise comparisons among the 7 dpa treatments revealed predominant downregulation: DMSO vs. APO (296 up/417 down), DMSO vs. H_2_O_2_ (251 up/342 down), and APO vs. H_2_O_2_ (136 up/109 down). Subsequent GO enrichment analysis categorized the DEGs into the three GO domains—cellular component, molecular function, and biological process—highlighting significant enrichment in biological processes and signaling pathways associated with ROS-mediated regeneration (Fig. S1). This functional profile was further summarized in a circular plot comparing the top eight enriched terms from each domain between control and treated groups.

### Pharmacological inhibition of NADPH oxidase-derived ROS impairs regeneration by suppressing immune and proliferative networks

**Fig. 4:**
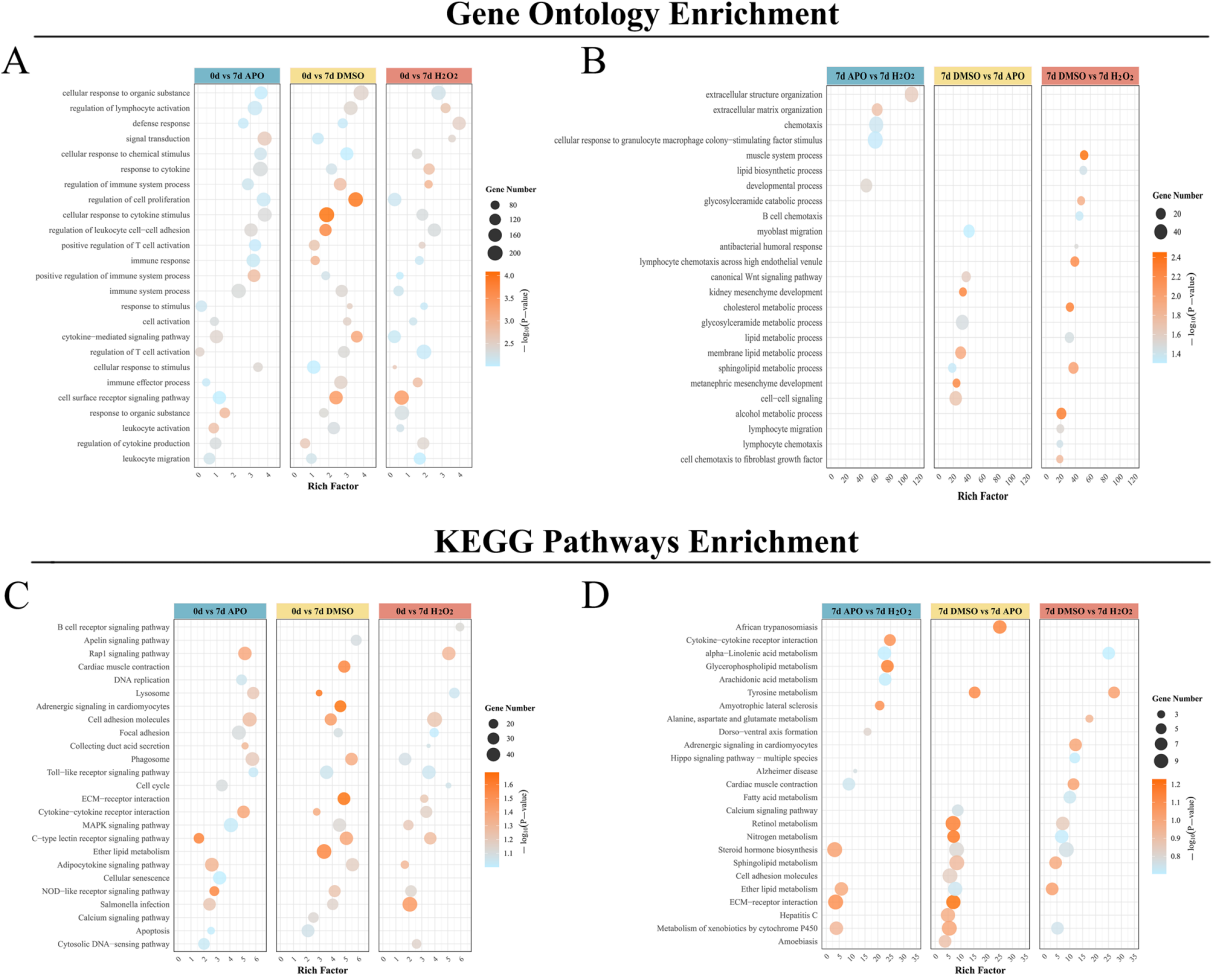
Gene set enrichment analysis of differentially regulated processes and pathways during tail regeneration. (**A**) Enriched biological processes and (**C**) pathways in tails from 0 dpa, 7 dpa APO-, and H_2_O_2_-treated groups. (**B**) Enriched biological processes and (**D**) pathways in tails from 7 dpa DMSO-, APO-, and H_2_O_2_-treated groups

Early regeneration (0 vs. 7 dpa, Control) is defined by a coordinated shift from structural to immune-proliferative programs (Fig. 4A, B). This shift involves significant upregulation of defense responses (GO:0006955, GO:0006954), cytokine-cytokine receptor interaction (ko04060), and MAPK signaling (ko04010), involving genes like *CD40* (DN42979_c0_g1), *CCL14* (DN4817_c3_g1), and *AKT1* (DN9344_c0_g1), alongside the downregulation of muscle function pathways (GO:0006936, GO:0055002). Conversely, pharmacological intervention reveals a redox-dependent regulatory axis (Fig. 4A-D). APO treatment severely disrupts the endogenous regenerative program. While it aberrantly enhances inflammatory pathways (ko04060, ko04145), it broadly suppresses key regenerative networks, including muscle development (GO:0051146), MAPK/Apelin signaling (ko04010, ko04371), and the expression of critical factors such as *FHL1* (DN456_c0_g1), *VEGFC* (DN10375_c0_g1), and *FGFR4* (DN11697_c0_g1). In contrast, H_2_O_2_ treatment amplifies pro-regenerative signaling, synergistically upregulating immune-inflammatory responses, cytokine activity (ko04060), and proliferative genes (*AKT1*,* PCNA*,* IL-6* family), while fine-tuning morphogenesis (GO:0048646) and ECM organization (GO:0030198). At 7 dpa, H_2_O_2_ enhances metabolic reprogramming (sterol biosynthesis, GO:0006695), structural remodeling (muscle contraction, ko04260; ECM-receptor interaction, ko04512), and drives Hippo-mediated proliferation via *TEAD1* (DN553_c2_g1) upregulation (Fig. 4C, D). Thus, H_2_O_2_ and APO elicit fundamentally opposing molecular responses, establishing ROS levels as a critical determinant of regenerative capacity through the integrated control of immune activation, metabolic reprogramming, and proliferative signaling.

### H_2_O_2_ coordinates top KEGG pathways to direct regenerative fate

#### Expression dynamics of phagosome pathway genes

**Fig. 5:**
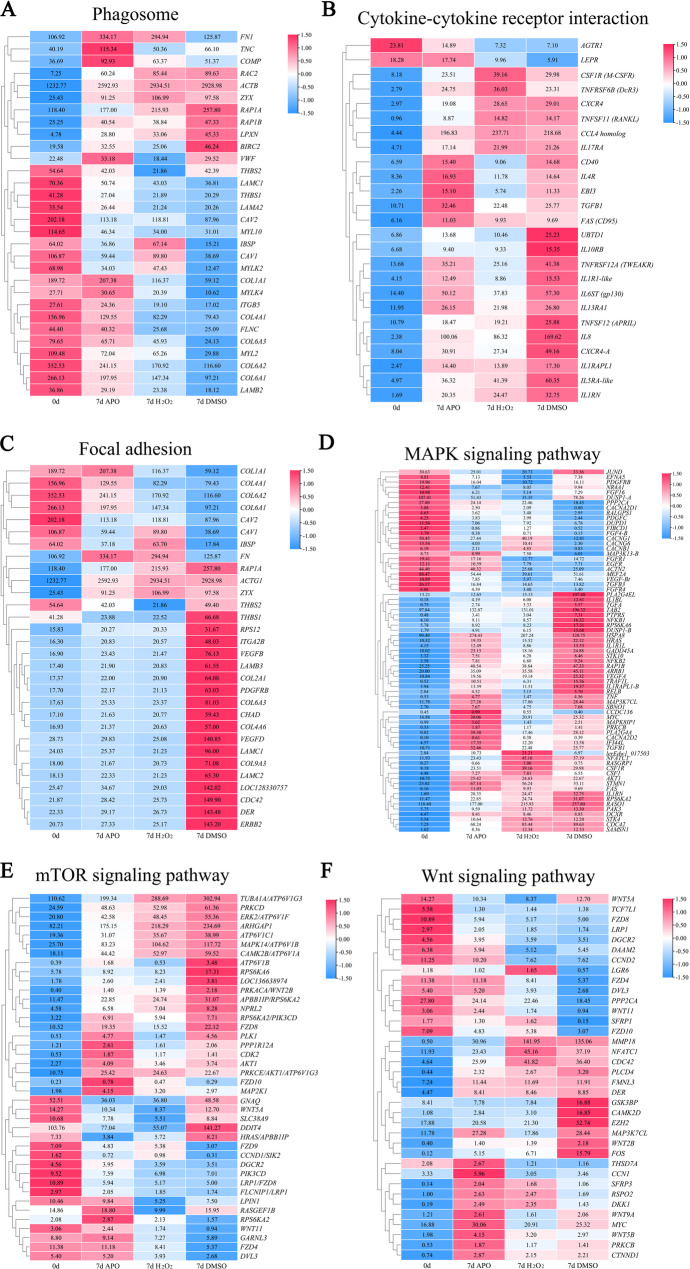
Pathway enrichment analysis of differentially expressed genes during tail regeneration under redox modulation. Heatmaps illustrate the expression patterns of (**A**) 30 phagosome-related genes, (**B**) 25 cytokine-cytokine receptor interaction network genes, (**C**) 30 focal adhesion pathway genes, (**D**) 71 MAPK signaling pathway genes, (**E**) 40 mTOR pathway genes, and (**F**) 36 Wnt signaling pathway genes across regeneration time points under DMSO, APO, and H_2_O_2_ treatments. All genes were identified via KEGG pathway analysis

The phagosome pathway analysis at 7 dpa reveals critical differences in the expression of genes governing debris clearance and matrix remodeling (Fig. 5A). *RAC2*, a master regulator of actin polymerization for phagocytic cup formation, was expressed at 85.44 ± 40.22 in the H_2_O_2_-treated group, significantly higher than the 60.24 ± 43.54 observed in the APO-treated group. This superior upregulation in the H_2_O_2_ group correlates with a more robust initiation of the engulfment phase. In contrast, the APO-treated group displayed a transcriptional signature indicative of a pro-fibrotic extracellular matrix (ECM) state. *COL1A1* expression remained elevated at 207.38 ± 92.54 in the APO group compared to 116.37 ± 72.15 in the H_2_O_2_ group. Similarly, *COL6A1* levels were higher in APO-treated animals (197.95 ± 89.11) versus H_2_O_2_-treated ones (147.34 ± 83.11). The DMSO vehicle control group showed intermediate expression, with *FN1* at approximately 125.87, compared to the higher levels in both treatment groups (APO: 334.17 ± 152.67; H_2_O_2_: 294.94 ± 131.24).

### Orchestration of the cytokine-cytokine receptor interaction network

Analysis of the cytokine-receptor interaction pathway demonstrates H_2_O_2_’s role as a conductor of a synchronized immune response (Fig. 5B). Key chemokines for immune cell recruitment were potently induced in the H_2_O_2_ group. The macrophage chemoattractant *CCL4* was expressed at 237.71 ± 143.49, surpassing the level in the APO group (196.83 ± 243.03). The neutrophil chemoattractant IL8 also showed peak induction in the H_2_O_2_ group (93.57 ± 41.43) compared to the APO group (53.82 ± 61.92). Concurrently, H_2_O_2_ enhanced the expression of cytoprotective factors, creating a balanced inflammatory milieu. The anti-apoptotic decoy receptor *TNFRSF6B* (*DcR3*) was most strongly induced in the H_2_O_2_ group (36.03 ± 22.37) versus the APO group (24.75 ± 16.11). A notable dysregulation was observed for *TGFB1*, which showed its highest expression in the APO group (32.46 ± 12.63), significantly above the H_2_O_2_ group (22.48 ± 3.39) and the control (10.71 ± 2.87).

### Regulation of focal adhesion complexes and cell motility

The focal adhesion pathway data show that H_2_O_2_ treatment significantly upregulates genes essential for cell migration and matrix adhesion (Fig. 5C). *ACTG1* (cytoplasmic actin) increased from a 0d baseline of 1,232.77 to 2,934.51 in the H_2_O_2_ group, a 2.4-fold induction. Zyxin (*ZYX*), critical for adhesion complex assembly, was upregulated 4.2-fold, from 25.43 to 106.99. *RAP1A*, regulating integrin signaling, increased 1.8-fold from 118.40 to 215.93.

### ROS coordinates a multi-pathway network to drive stage-specific tail regeneration

#### MAPK pathway dynamics correlate with early epithelial closure

The MAPK pathway was significantly enriched across all groups (*p* < 1.2 × 10^− 8^) (Fig. 5D). In controls, epithelialization at 7 dpa was associated with elevated expression of upstream regulators *HSPA8* (144.44 ± 35.93) and *RASO1* (118.40 ± 15.01). APO impaired this process, with *CDC42* (23.45 ± 10.18) and *HRAS* (10.52 ± 1.11) expression significantly lower than control (*p* < 0.05), coinciding with delayed epithelial coverage and persistent inflammation. Exogenous ROS (H_2_O_2_) accelerated wound closure, correlating with markedly higher *CDC42* expression (85.77 ± 26.76, *p* < 0.01) and downregulation of the MAPK phosphatase *DUSP1-A* (to 0.4-fold). Branch-specific MAPK activation underlies phenotypic divergence: APO favored ERK-branch activation, upregulating *HRAS*,* RAP1B*, and *MAP3K7CL* by 1.8-2.5-fold, along with *JUND* and *MYC* (both increased by 1.9-fold), while H_2_O_2_ strongly activated the p38 branch (average increased by 2.8-fold) and downregulated *JUND* to 0.7-fold (*p* < 0.01). Network analysis confirmed distinct connectivity patterns: APO enriched an ERK→JUND/MYC→proliferation network (NFATC1 connectivity = 48), whereas H_2_O_2_ activated a p38→ATF2→stress-response network (NFATC1 connectivity = 32).

### mTOR and Wnt pathways are reprogrammed in a ROS-dependent manner

mTOR signaling exhibited a biphasic response (Fig. 5E), characterized by early upregulation of the mTORC1 inhibitor *DDIT4* (4.7-fold increase in 7 dpa-H_2_O_2_ versus 0 dpa) followed by induction of the mitotic regulator *PLK1* (18.5-fold increase in 7 dpa compared to 0 dpa). Wnt signaling displayed functional bifurcation expression of a homeostatic module (including *WNT5A*,* WNT11*,* DVL3*,* FZD4*) was progressively suppressed after amputation (Fig. 5F), with the highest level observed at 0 dpa, followed by 7 dpa-APO, and the lowest levels in 7 dpa and 7 dpa-H_2_O_2_ groups. Conversely, a pro-regenerative module (including *CCND2*,* MYC*,* FZD8*,* FZD10*,* RSPO2*) was robustly activated in a ROS-dependent manner, showing strongest activation in 7 dpa and 7 dpa-H_2_O_2_ groups, moderate activation in 7 dpa-APO, and weakest activation at 0 dpa.

### Hippo-YAP pathway is dynamically regulated by ROS

**Fig. 6:**
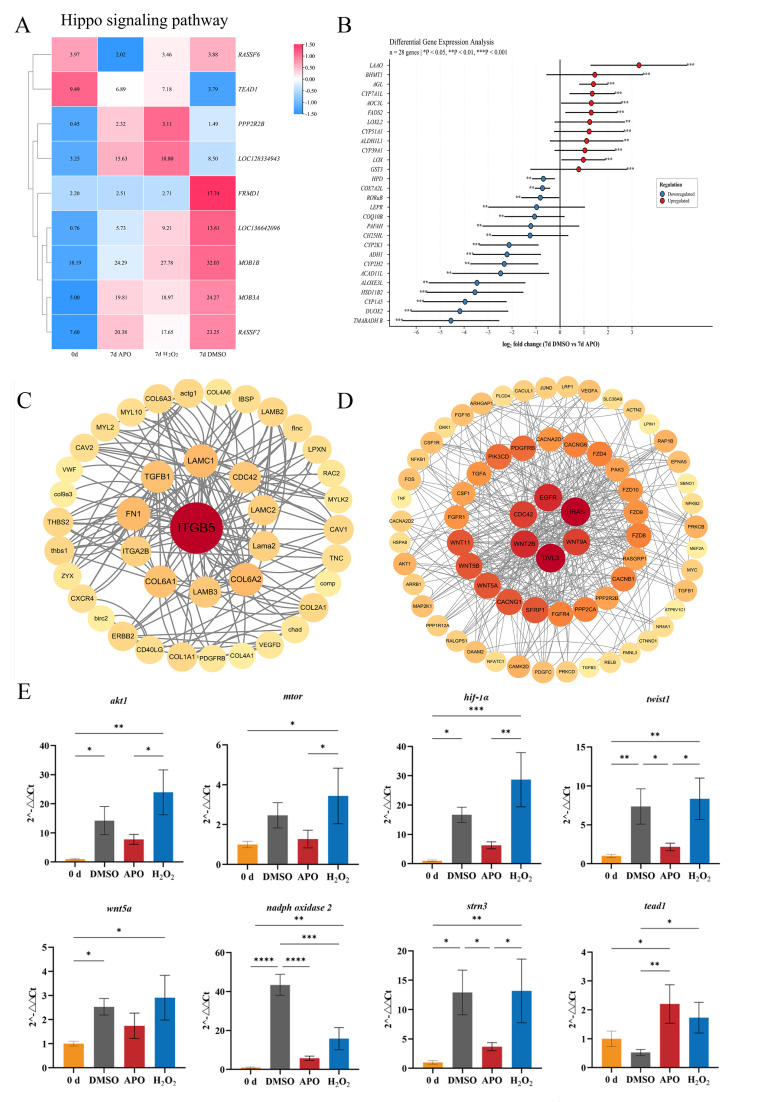
Transcriptome profiling and network analysis of redox-regulated signaling during *S. tsinlingensis* tail regeneration at 7 dpa. (**A**) Heatmap of 9 differentially expressed Hippo-related genes across regeneration time points. (**B**) Forest plot of 28 redox-related genes (APO vs. DMSO at 7 dpa, *p* < 0.05) shows downregulation of ROS-generating enzymes (*DUOX2*, *CYP* isoforms, *ALOXE3L*) and upregulation of *BHMT1*. (**C**) Protein interaction network centered on ITGB5 integrates extracellular matrix components (COL/LAM, FN1) with signaling molecules (TGFB1, PDGFRB) to coordinate matrix adhesion and intracellular signaling. (**D**) Multi-hub regulatory network for wound closure and blastema formation, with core hubs (HRAS, EGFR, DVL3, WNT2B, WNT9A) forming interconnected Wnt and EGFR-RAS-MAPK pathways linked via microenvironment sensors (PDGFRB, CSF1) to modules controlling epithelial migration, blastema dedifferentiation, and tissue remodeling. (**E**) qRT-PCR validation confirms RNA-seq results: H_2_O_2_ upregulates *hif1a*, *twist1*, and *tead1*, while APO downregulates *nadph oxidase 2* and *strn3* (*n* = 3 for each group), consistent with enhanced YAP inactivation

In controls, *MOB1B* increased from 18.19 to 32.03, while *TEAD1* decreased from 9.49 to 3.79, and *RASSF2* rose from 7.60 to 23.25, correlating with normal epithelialization (7 dpa) and blastema formation (14 dpa). Under ROS inhibition (APO), *MOB1B* rose only to 24.29, *TEAD1* decreased to 6.89, and *RASSF6* declined from 3.97 to 2.02, matching delayed epithelialization and persistent inflammation. With exogenous ROS (H_2_O_2_), *MOB1B* reached 27.78, *TEAD1* fell to 7.18, and *LOC1*36642096 surged from 0.76 to 9.21, consistent with accelerated epithelial coverage by 7 dpa and early blastema appearance by 14 dpa (Fig. 6A).

Integrated transcriptomic and pharmacological analyses demonstrate that reactive oxygen species (ROS) orchestrate the spatiotemporal regulation of MAPK, mTOR, Wnt, and Hippo-YAP signaling pathways, which collectively dictate the efficiency of wound epithelialization and blastema formation during tail regeneration.

### APO attenuates enzymatic ROS production through multi-target inhibition

To investigate the effect of APO treatment on the expression profile of redox-related genes, the differential expression patterns between 7d APO and 7d DMSO groups were analyzed using a forest plot (*p* < 0.05). Transcriptomic analysis revealed that APO treatment significantly altered the expression profile of redox-related genes (Fig. 6B). Among the 28 genes examined, 22 (78.6%) showed statistically significant differential expression (*p* < 0.05). Notably, the expression of *DUOX2*, a key enzyme responsible for programmed reactive oxygen species (ROS) production, was markedly suppressed (*p* < 0.01). This inhibitory effect occurred in concert with the downregulation of multiple other redox-associated genes, including cytochrome P450 family members (e.g., *CYP1A5*,* CYP2H2*, *p* < 0.001) and the lipoxygenase *ALOXE3L* (*p* < 0.05). Concurrently, the antioxidant and methylation-related gene *BHMT1* exhibited the most substantial upregulation (log2FC = + 1.45, *p* < 0.001). Collectively, these expression changes indicate that APO simultaneously targets multiple critical sources of enzymatic ROS generation.

### Stage-specific protein interaction networks underlie regenerative coordination

To delineate stage-specific signaling crosstalk during tail regeneration, we conducted sequential protein-protein interaction (PPI) network analyses. First, to resolve mechanisms underlying cell–matrix interactions, a PPI network was constructed from differentially expressed genes (DEGs) enriched in the top three KEGG pathways: phagosome, cytokine–cytokine receptor interaction, and focal adhesion. This network (Fig. 6C), comprising over 50 proteins, revealed a centralized architecture with ITGB5 (integrin subunit beta 5) as the sole core hub (deep-red node). ITGB5 directly interacted with intermediate orange-core proteins—FN1 (fibronectin 1), ITGA2B (integrin subunit alpha 2b), and LAMC1 (laminin subunit gamma 1)—which connected further to peripheral light-yellow nodes, organizing into three cohesive modules: an extracellular matrix (ECM) remodeling module (centered on the COL [collagen] and LAM [laminin] families), a cell-adhesion module (mediated by FN1 and ITGA2B), and a signal-transduction module (involving TGFB1 [transforming growth factor beta 1] and PDGFRB [platelet-derived growth factor receptor beta]). Functionally, ITGB5 serves as the integrin nexus, both anchoring cell–matrix contacts by binding ECM components and transducing extracellular cues via TGFB1/PDGFRB signaling, thereby synchronizing adhesion, remodeling, and transduction to facilitate cell migration and proliferation at this stage.

To elucidate the signaling landscape during the earlier wound epithelium and blastema initiation phase, a distinct PPI network was built from 64 proteins encoded by DEGs concurrently enriched in four pro-regenerative pathways: MAPK, mTOR, Wnt, and Hippo-YAP (|log2FC| > 1.5, pathway enrichment *p* < 0.001). This network displayed a multi-hub, coordinated architecture, with deep-red core nodes (HRAS, EGFR, DVL3, WNT2B, WNT9A) integrating key pathways (Fig. 6D). Here, DVL3 mediated Wnt signaling through interactions with multiple Wnt ligands, while EGFR and HRAS constituted the core MAPK/proliferation axis. Orange intermediary nodes (e.g., WNT11, WNT5B, PDGFRB, CSF1) extended connectivity to peripheral proteins (e.g., FGFs, MMPs, ACTs), defining three functional modules: epithelial migration/proliferation (centered on EGFR/FGFs/MAPK), blastema dedifferentiation (driven by DVL3-Wnt), and microenvironment remodeling (via PDGFRB/CSF1/mTOR-related signals). This organization establishes a “dual-pathway core plus microenvironment regulation” paradigm, wherein crosstalk between the Wnt and EGFR-RAS-MAPK pathways, underpinned by PDGFRB/CSF1-mediated microenvironmental input, synchronizes wound epithelialization, cell dedifferentiation, and tissue adaptation to molecularly prime subsequent blastema progression.

### qRT-PCR validation confirms key transcriptomic findings

Based on transcriptomic sequencing results, eight genes—*akt1*,* mtor*,* hif1a*,* twist1*,* wnt5a*,* nadph oxidase 2*,* strn3*,* and tead1*—were selected for qRT-PCR analysis (Fig. 6E), with gapdh serving as the internal reference gene. Compared to the 0 d group, *akt1* expression was significantly upregulated in the DMSO group, consistent with transcriptomic data. The H_2_O_2_ group exhibited markedly higher *hif1a* transcription than the APO group (*p* < 0.01), corroborating RNA-seq trends. Similarly, *twist1*, a downstream target of *hif1a*, showed significantly elevated expression in H_2_O_2_ versus APO treatments (*p* < 0.01). In the APO group, *strn3* was significantly downregulated (*p* < 0.05), promoting YAP phosphorylation and aligning with transcriptomic findings. ROS-related genes *duoxa2* and *nadph oxidase 2* were strongly suppressed in APO-treated samples compared to controls (*p* < 0.0001). Conversely, *tead1* expression was significantly higher in H_2_O_2_ versus DMSO groups (*p* < 0.01), consistent with RNA-seq results. Overall, qRT-PCR validation confirmed the reliability of the transcriptomic data.

### Spatiotemporal immunofluorescence analysis reveals H_2_O_2_-regulated signaling networks during tail regeneration

**Fig. 7:**
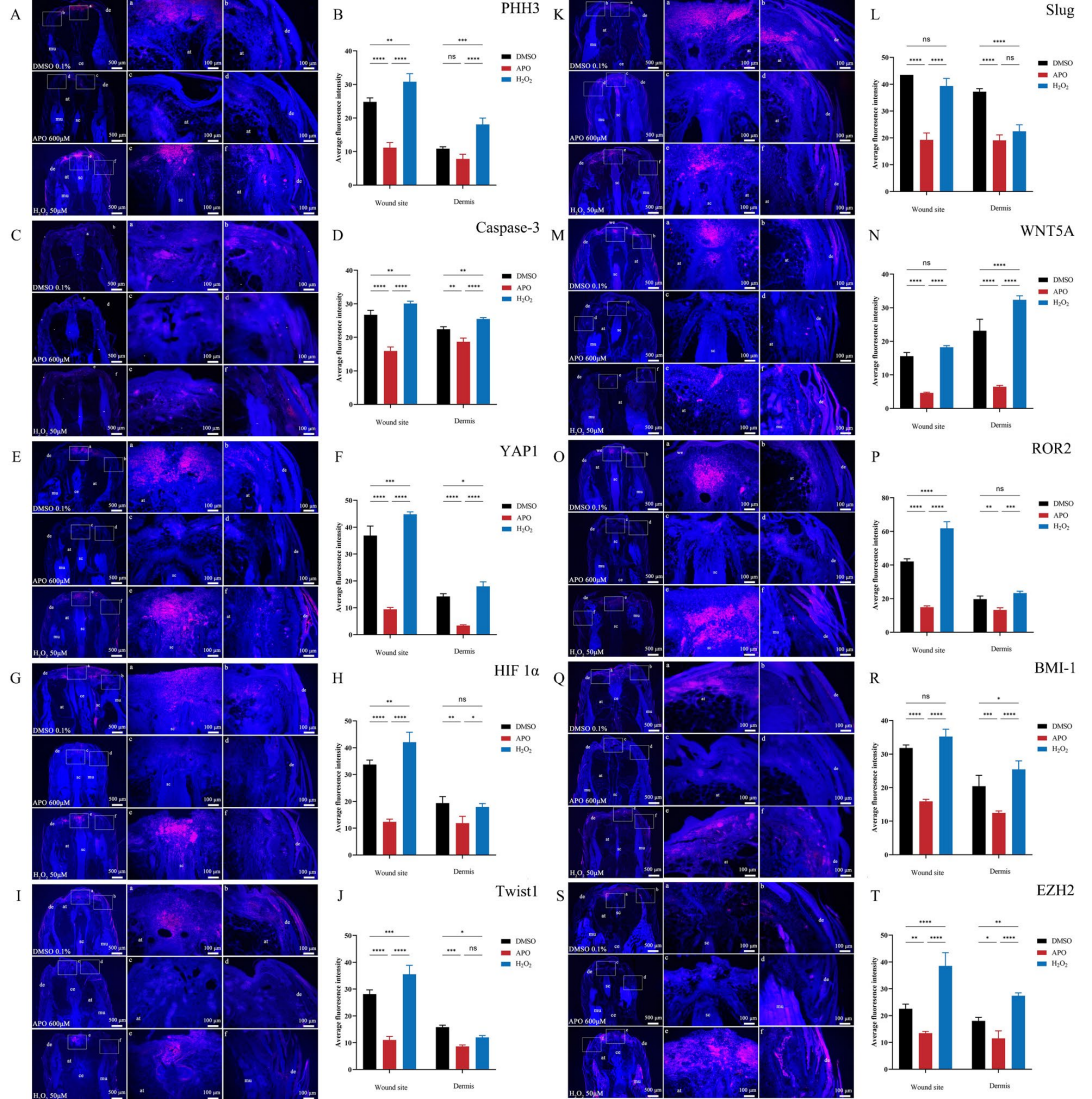
H_2_O_2_ promotes cell proliferation, suppresses apoptosis, and activates key regenerative pathways in regenerating *S. tsinlingensis* tails at 7 dpa. Immunofluorescence analysis shows H_2_O_2_ elevates PHH3 (**A**, **B**) and reduces Caspase-3 (**C**, **D**), while APO exhibits opposing effects on Caspase-3. H_2_O_2_ enhances expression of YAP1 (**E**, **F**), blastema-specific HIF-1α (**G**, **H**) and Twist1 (**I**, **J**), and differentially regulates Slug (**K**, **L**). H_2_O_2_ maintains WNT5A (**M**, **N**) and upregulates ROR2 at wound sites (**O**, **P**), whereas APO suppresses these markers. Both BMI-1 (**Q**, **R**) and EZH2 (**S**, **T**) are upregulated by H_2_O_2_ and downregulated by APO. All panels display representative images (left) and mean fluorescence intensity quantifications (right) in wound/blastema and dermal regions (*n* = 3 for each group). Scale bars: 200 μm. Abbreviations: at, adipose tissue; ce, central vertebra; de, dermis; mu, muscle; sc, spinal cord. Data are mean ± SD. Low-magnification images and corresponding high-magnification images (Ab, Ad, Kb, Kd) were obtained from different individuals within the same experimental group, each selected for optimal representation of tissue morphology (low-magnification) or fluorescent labeling clarity (high-magnification). All key structural features were consistent across biological replicates

Immunostaining for phosphorylated histone H3 (PHH3) demonstrated that H_2_O_2_ treatment significantly increased PHH3 fluorescence intensity in both the blastema (30.79 ± 2.40) and dermis (18.08 ± 1.89) compared to controls, whereas APO treatment markedly reduced PHH3 expression in the blastema (11.13 ± 1.56) (Fig. 7A, B). In parallel, H_2_O_2_ increased Caspase-3 activity in the wound (30.08 ± 0.69) and dermal regions (25.45 ± 0.46), while APO decreased Caspase-3 levels (Fig. 7C, D). These results indicate that H_2_O_2_ coordinately regulates proliferation and apoptosis during blastema formation. Immunostaining showed that H_2_O_2_ elevated YAP1 protein levels in wound (44.82 ± 0.90) and dermal tissues (18.01 ± 1.65), whereas APO strongly suppressed YAP1 expression (Fig. 7E, F). Similarly, H_2_O_2_ increased HIF-1α expression specifically in the blastema (42.05 ± 3.73), while APO reduced HIF-1α levels in both regions (Fig. 7G, H). These findings suggest that ROS promote regenerative signaling via the Hippo-YAP pathway and hypoxia adaptation. ROS influence EMT-related transcription factors. H_2_O_2_ treatment increased Twist1 protein expression in the blastema (35.54 ± 3.31), whereas APO reduced Twist1 in both the blastema and dermis (Fig. 7I, J). Slug expression was also decreased by APO, while H_2_O_2_ caused a region-specific reduction in dermal Slug (Fig. 7K, L). These data imply that ROS support epithelial-mesenchymal transition during regeneration. WNT5A/ROR2 signaling is redox-sensitive. APO treatment suppressed both WNT5A (Fig. 7M, N) and its receptor ROR2, while H_2_O_2_ maintained or increased their expression, particularly elevating ROR2 in wound areas (61.83 ± 3.88) (Fig. 7O, P). This indicates that ROS help sustain non-canonical Wnt signaling, which may contribute to cell cycle regulation during regeneration. Epigenetic regulators BMI-1 (Fig. 7Q, R) and EZH2 (Fig. 7S, T) are redox-modulated. H_2_O_2_ enhanced the expression of chromatin modifiers BMI-1 (dermis: 35.21 ± 2.21) and EZH2 (wound: 38.53 ± 4.97; dermis: 27.39 ± 1.10), while APO reduced their levels. These results demonstrate that ROS influence epigenetic remodeling during tissue regeneration.

Immunofluorescence analyses reveal that H_2_O_2_ promotes a pro-regenerative state by enhancing proliferation, activating YAP1 and HIF-1α, modulating EMT markers and Wnt signaling, and upregulating epigenetic regulators. Conversely, ROS inhibition by APO disrupts these coordinated processes. Together, these findings establish ROS as a central coordinator of the cellular and molecular events required for successful tail regeneration.

## Discussion

The successful regeneration of complex structures like the lizard tail represents a remarkable biological process governed by highly conserved molecular mechanisms. Our study demonstrates that ROS, particularly H_2_O_2_, function as indispensable master regulators coordinating a multi-pathway network to drive tail regeneration in *S. tsinlingensis*. The contrasting phenotypes observed—accelerated healing with H_2_O_2_ treatment versus significant delay with the NADPH oxidase inhibitor apocynin (APO)—directly reflect the success or failure of this intricate, ROS-dependent coordination.

### The evolutionary conservation of NADPH oxidase-dependent ROS signaling

Our findings reinforce the fundamental principle that NADPH oxidase (Nox)-mediated ROS production is an evolutionarily conserved cornerstone of regenerative capacity across vertebrates. In *S. tsinlingensis*, *Nox2* and *DUOXA2* contribute to ROS generation, aligning with the essential roles of Nox homologs in salamanders [14], geckos [46], Xenopus [47], and zebrafish [48]. The phenotypic parallels are striking: APO inhibition delayed epidermal coverage and blastema formation, mirroring defects in APO/VAS2870-treated salamanders [14] and axolotls. This conservation extends to therapeutic rescue, where H_2_O_2_ promoted regeneration and rescued APO-induced inhibition, consistent with H_2_O_2_-mediated rescue in multiple models, including Xenopus refractory period regeneration [13] and Nox-inhibited salamander larvae [14]. Recent work in zebrafish further underscores this conservation, demonstrating that ROS counteract wound contraction to promote healing [49]. In that study, acute ROS inhibition (DPI/NAC) or chronic duox knockdown led to prolonged contraction, delayed closure, and impaired regrowth—phenotypes analogous to our APO-induced defects. Critically, Ding et al. (2025) showed that exogenous H_2_O_2_ supplementation rescued overcontraction and promoted wound relaxation and closure, reinforcing H_2_O_2_’s role as a critical, conserved redox signal for tissue repair [49].

### ROS orchestrates blastema formation by coordinating proliferation, apoptosis, and tissue mechanics

Blastema formation necessitates a precise balance of cellular processes. Our data show sustained H_2_O_2_ administration promoted blastemal accumulation and pcna upregulation, consistent with its role in restoring proliferation in Nox-inhibited axolotl blastemas [14]. This proliferative effect is conserved, as exogenous H_2_O_2_ restores Nox activity and cell cycling in multiple models [48, 12]. Furthermore, H_2_O_2_ enhanced Caspase-3 activation alongside blastemal growth, supporting the model of ROS-dependent, apoptosis-induced compensatory proliferation—a mechanism observed in Drosophila [50], hydra, and zebrafish [51, 52].

Beyond cell number control, ROS critically modulate the biomechanical environment. The role of ROS in fine-tuning cellular mechanics is highlighted by Ding et al. (2025), who found that ROS suppress phosphorylation of myosin regulatory light chain (p-MRLC), thereby relaxing the actomyosin ring and preventing overcontraction [52]. This mechanical relaxation facilitated wound closure without requiring actomyosin-driven contraction, aligning with the idea that ROS create a permissive cellular environment for regeneration. Moreover, chronic ROS depletion in zebrafish altered tissue stiffness and epithelial morphology [52], similar to how APO disrupted blastemal architecture in our model. Their use of atomic force microscopy and computational simulations revealed that reduced tissue rigidity contributed to dysfunctional contraction—a concept crucial for understanding how ROS modulate the biomechanical landscape during regeneration.

### ROS as a spatiotemporal coordinator of an integrated signaling network

Our transcriptomic and phenotypic analyses reveal that H_2_O_2_ acts as a master temporal and spatial coordinator, synchronizing multiple core pathways into a coherent regenerative program. Early Phase Coordination: The early phase (0–7 dpa) requires ROS-dependent activation of specific MAPK signaling. H_2_O_2_ enhanced *CDC42* expression and suppressed *DUSP1*, prolonging p38/MAPK signaling to accelerate wound closure and epithelial migration. APO disrupted this early signal, leading to persistent inflammatory-like cell infiltration and delayed epithelialization despite later compensatory upregulation of ERK-associated proliferative genes (*JUND*, *MYC*), highlighting the critical importance of precise timing in pathway activation.

Immune and Microenvironment Modulation: Transcriptome analysis revealed peak immune activation at 7 dpa under H_2_O_2_ treatment, with upregulation of chemokines and receptors that promote immune cell migration. H_2_O_2_ orchestrated a balanced immune response—co-inducing recruitment signals (*CCL4*, *IL8*) and protective factors (*DcR3*)—while APO promoted a pro-fibrotic signature (elevated *TGFB1*). This aligns with established roles of ROS in leukocyte recruitment during zebrafish tail [15] and axolotl limb regeneration [14]. However, the context-dependence of inflammation is evident, as immune cell depletion does not impede fin regeneration in zebrafish [53], suggesting ROS’s primary regenerative function extends beyond immune activation.

Integration with Developmental Pathways: ROS integrate with key developmental hubs. We found ROS inhibition reduced YAP1-positive cells and upregulated inhibitory Hippo pathway genes (*frmd1*, *map4k1*, *fat4*), aligning with observations that ROS regulate YAP1 activity in cancer models [54, 55] and its conserved role in appendage regeneration [56–58]. Simultaneously, the *ROS-HIF-1α* axis coordinated epithelial-mesenchymal transition (EMT) and cell cycle regulation. H_2_O_2_ activated HIF-1α signaling and promoted EMT markers (*Twist1*, *Slug*), consistent with *HIF-1α*’s role in driving EMT. This axis likely regulates the conserved G2/M arrest observed in regenerative blastemas across species [59, 60]. Metabolic and transcriptional reprogramming: ROS orchestrate a metabolic switch via mTOR inhibition and Wnt pathway modulation. The rapid induction of *DDIT4* and suppression of homeostatic Wnt genes (*WNT5A*, *WNT11*) create a dedifferentiated, permissive state necessary for blastema formation. H_2_O_2_ enhanced this reprogramming, leading to early blastema appearance, while APO attenuated both *DDIT4* upregulation and homeostatic Wnt suppression, delaying the metabolic switch.

While this study establishes the ROS-MAPK-mTOR-Wnt axis as a critical regulator, several questions remain. The dual role of ROS requires clarification through genetic approaches, such as targeted knockout of ROS-generating enzymes, to define precise source-specific functions. Transcriptomic insights should be integrated with proteomic analyses to fully elucidate the molecular mechanisms. Future work must also delineate how ROS interfaces with other key pathways, particularly the Hippo-YAP pathway, and elucidate the molecular basis of ROS-mediated epithelial-mesenchymal transition (EMT) induction.

While this study, based on transcriptomic and pharmacological data, suggests a critical regulatory role for H_2_O_2_ in tail regeneration, several questions remain. The dual role of ROS requires clarification through genetic approaches, such as targeted knockout of ROS-generating enzymes, to define precise source-specific functions. Transcriptomic insights should be integrated with proteomic analyses to fully resolve the underlying molecular mechanisms. Future work must also delineate how ROS interfaces with other key pathways, particularly the Hippo-YAP pathway, and directly validate the suggested link between ROS and epithelial-mesenchymal transition (EMT).

## Concluding remarks

Based on transcriptomic and pharmacological data, this study suggests that H_2_O_2_ acts as a critical regulator of tail regeneration in *S. tsinlingensis*, driving distinct transcriptional programs with minimal overlap (only five shared DEGs between DMSO-vs-APO [713 DEGs] and DMSO-vs-H_2_O_2_ [593 DEGs]) and coordinating an integrated repair network through sequential activation of MAPK, mTOR, Wnt, and Hippo-YAP signaling. The presence of a time-sensitive, H_2_O_2_-dependent plasticity window explains limited rescue upon delayed intervention. Consistent with vertebrate models [49], our findings support the paradigm that NADPH oxidase-derived ROS function as spatiotemporal integrators of tissue repair, orchestrating injury response, immune modulation, metabolic adaptation, and morphogenesis. Pharmacological ROS inhibition via APO appears to involve dual suppression of enzymatic ROS sources and enhancement of methylation-dependent antioxidant defense, reprogramming redox homeostasis to delay regeneration. These findings highlight spatiotemporal ROS dynamics as a potential therapeutic target in wound healing, while future studies should employ genetic and multi-omics approaches to directly validate the inferred associations, including context-specific ROS functions, pathway crosstalk, and the suggested link between ROS and EMT.

## Supplementary Information

Below is the link to the electronic supplementary material.

Supplementary Material 1

## Data Availability

No datasets were generated or analysed during the current study.
