# Supplementary material for: Reactive oxygen species drive wound epithelium and blastema formation in regenerating tail of Scincella tsinlingensis
Source: Front Zool. 2026 Jun 2;23:29. doi: 10.1186/s12983-026-00617-2 (PMC13455431; doi:10.1186/s12983-026-00617-2)

Supplemental Information

Supplementary Table S1 Results of transcriptome sequencing transcriptome data filtering and assembly

Sample	Read number	Base number	GC（%）	Q30（%）
0 d-1	22,630,437	6,674,481,198	48.56	94.49
0 d-2	23,190,743	6,843,051,916	48.72	94.38
0 d-3	24,063,822	7,025,779,516	48.43	94.55
7 d APO-1	22,859,731	6,758,087,084	48.55	94.34
7 d APO-2	21,962,253	6,483,028,998	48.14	94.36
7 d APO-3	21,048,166	6,204,845,338	48.82	94.41
7 d H_2O_2-1	22,246,877	6,581,125,602	48.28	94.42
7 d H_2O_2-2	25,010,088	7,410,468,559	47.82	94.42
7 d H_2O_2-3	22,431,270	6,619,567,366	48.52	94.37
7 d-1	22,983,926	6,751,353,107	48.44	94.63
7 d-2	22,167,344	6,538,324,131	47.77	94.44
7 d-3	22,539,458	6,643,872,335	48.07	94.20

Supplementary Table S2 Data assembly results

Length Range	Transcript	Unigene
200-300	33,457（18.22%）	27,488（27.73%）
300-500	35,856（19.52%）	25,456（25.68%）
500-1000	36,083（19.65%）	21,043（21.23%）
1000-2000	32,053（17.45%）	11,657（11.76%）
2000+	46,207（25.16%）	13,476（13.60%）
Total Number	183,658	99,121
Total Length	277,951,515	101,757,689
N50 Length	3,009	2,253
Mean Length	1513.42	1026.6

Supplementary Table S3 Results of Unigene annotation

Anno_Database	Annotated_Number	length（bp）300-1000	length>=1000
COG_	4,520	707	3,446
GO	19,942	4,413	13,936
KEGG	19,434	4,296	13,489
KOG	14,006	2,445	10,643
Pfam	17,775	3,439	13,180
Swissprot	11,193	1,737	8,912
TrEMBL	24,481	6,285	15,637
eggNOG	21,354	5,181	14,221
NR	23,567	5,809	15,477
All Annotated	26,749	7,514	15,977

Supplementary Figure S1 GO enrichment map across the 0 d control, 7 d DMSO, 7 d APO, and 7 d H_2O_2 groups

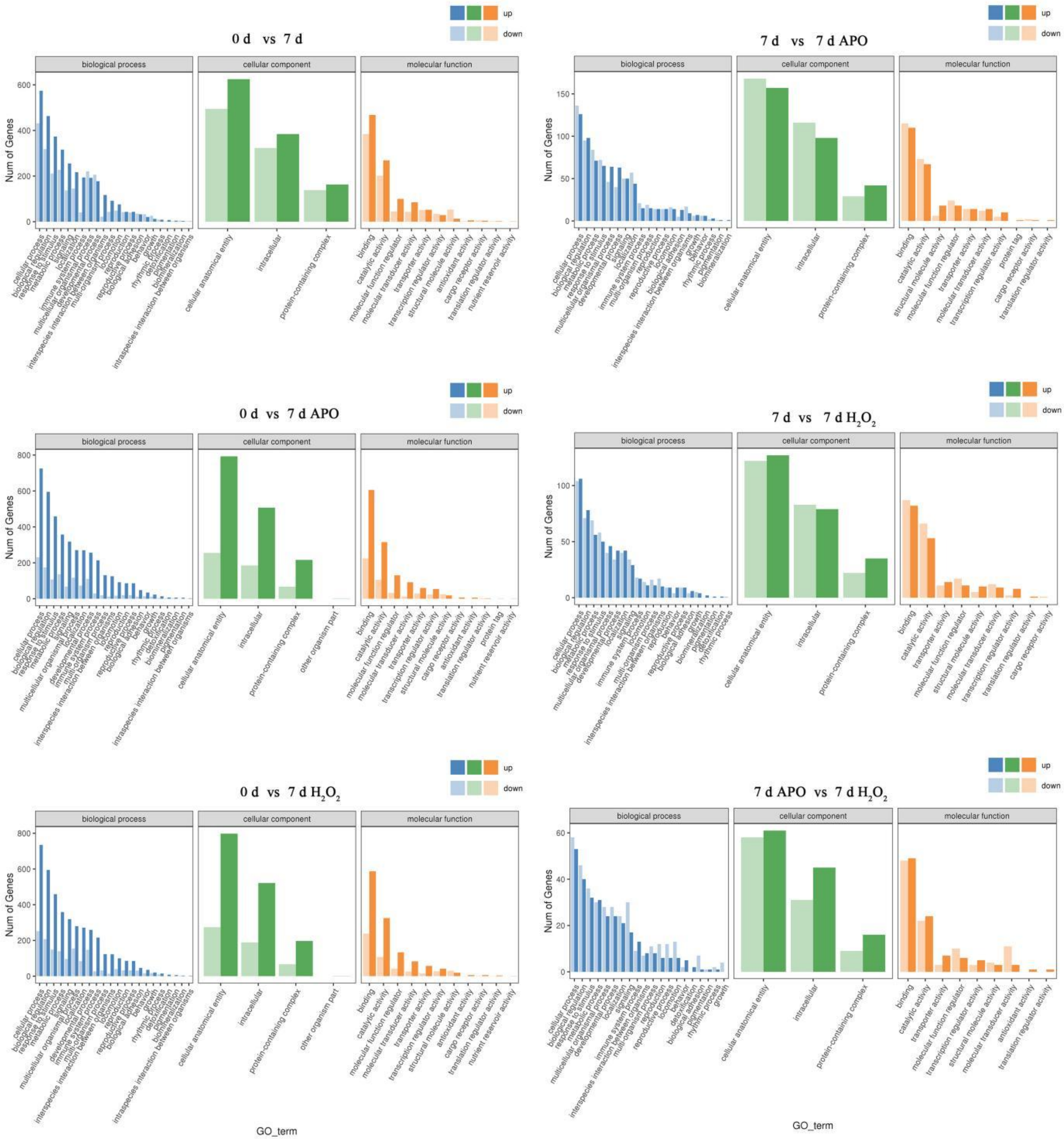

Supplement: Supplementary file 1 [file 12983_2026_617_MOESM1_ESM.pdf]
